# Acute Myopericarditis after COVID-19 Vaccine in Teenagers

**DOI:** 10.1155/2021/8268755

**Published:** 2021-09-20

**Authors:** Shashikanth Ambati, Michael Colon, Maya Mihic, Javier Sanchez, Adnan Bakar

**Affiliations:** ^1^Division of Pediatric Critical Care, Department of Pediatrics, Bernard & Millie Duker Children's Hospital, Albany, New York, USA; ^2^Pediatric Cardiology, Capital District Pediatric Cardiology Associates, Albany, New York, USA; ^3^Department of Pediatrics, Bernard & Millie Duker Children's Hospital, Albany, New York, USA

## Abstract

**Background:**

There have been an increasing number of reports of myocarditis and pericarditis in adolescents and young adults after coronavirus disease 19 vaccinations. The pathophysiology of myocarditis after this vaccination is indeterminate currently. The problem is a relatively new phenomenon, and so there are no current guidelines on how to manage these cases of myopericarditis. We intend to describe our management in these two cases so that it can help guide pediatricians, intensivists, and cardiologists taking care of similar cases. *Case Summaries*. The first case is a young adolescent who presented with chest pain after receiving his second dose of coronavirus disease 19 vaccination with no other symptoms. His troponin was found to be 40 ng/mL. He had a normal echocardiogram and chest CT angiogram. His troponins trended down with symptomatic pain management after 3 days. The second case is another adolescent who presented with fever, fatigue, headache, and chest pain 3 days after receiving his second dose of coronavirus vaccine. His troponin was elevated to 5 ng/mL, electrocardiogram with ST segment elevations, and mildly decreased systolic function on echocardiogram. His troponins and electrocardiogram were normalized in 3 days at the time of his discharge.

**Conclusion:**

The clinical course of vaccine-associated myocarditis appears favorable as both our patients have responded well to medications and rest with prompt improvement in symptoms with full recovery. The experience remains limited at this time regarding the investigations, management, and follow-up of this novel clinical entity. It is vital for all the health care providers taking care of adolescents to have knowledge about this phenomenon and make correct diagnosis in those presenting with chest pain after COVID-19 vaccine and in preventing unnecessary invasive procedures such as coronary angiogram to rule out acute coronary syndromes.

## 1. Introduction

There have been increased reported cases of myocarditis and pericarditis after the mRNA COVID-19 vaccines (Pfizer-BioNTech and Moderna) particularly in adolescent and young adults since April 2021 [1]. The Advisory Committee on Immunization Practices (ACIP) issued the recommendation for use of Pfizer-BioNTech COVID-19 vaccine in adolescents aged 12-15 years under the Food and Drug Administration's (FDA) Emergency Use Authorization (EUA) on May 12, 2021 [2]. More than 20 million adolescents and young adults have been vaccinated in the United States so far. Recently, the Center for Disease Control (CDC) confirmed 226 cases of myocarditis or pericarditis in people younger than 30 years of age who have received an mRNA COVID-19 vaccine [3]. Although rare, the case rates per million doses after second dose of the vaccine are higher than the expected for this age group. The numbers above were reported to vaccine adverse event reporting system (VAERS), and there are another 250 more reports that federal health officials are investigating. It is not yet clear whether these cases represent true myocarditis or pericarditis. The true incidence of myocarditis/pericarditis after COVID-19 vaccine may not be known as every case may not be reported.

Marshall et al. [4] recently published a case series of seven adolescents with myocarditis following Pfizer-BioNTech COVID-19 vaccine. Currently, there is no definite causal relationship of myocarditis with the COVID-19 vaccine. The pathophysiology of myocarditis after COVID-19 vaccine is indeterminate at this time. However, there have been multiple reports of myopericarditis associated with active COVID-19 infection, as a delayed complication in COVID-19 adult survivors, and as a part of multi-inflammatory syndrome in children (MIS-C) [5]. The pathogenesis of myocarditis or pericarditis associated with COVID-19 infection is thought to be the combination of direct viral injury or the host's immune response resulting in myocardial damage [6].

The problem is a relatively new phenomenon, and there are no current guidelines on how to manage these cases of myopericarditis. Here, we describe two adolescents who developed myopericarditis after receiving the second dose of Pfizer-BioNTech COVID-19 vaccine requiring intensive care unit admission followed by full recovery. At this time, there are not many case reports published about this condition in adolescents other than the reports to VAERS and some anecdotal reports. Hence, we intend to describe our management in these two cases so that it can help guide pediatricians, intensivists, and cardiologists taking care of similar cases.

## 2. Case 1

A 16-year-old male with history of Von Willebrand disease, anxiety disorder, and Lennox-Gastaut syndrome on multiple seizure medications and vagal nerve stimulator presented to the emergency department (ED) with chest pain 2 days after receiving his second dose of Pfizer-BioNTech COVID-19 vaccine. There was no associated shortness of breath, palpitations, or syncope. The chest pain was described as stabbing in nature, localized to the substernal area, and worsened with respirations. He also reported to have couple episodes of emesis the morning of presentation but denied any fevers, abdominal pain, diarrhea, or any breakthrough seizures. There was no known exposure or history of COVID-19 in the past. He initially presented to a local urgent care after his chest pain got worsened. His electrocardiogram (ECG) demonstrated diffuse ST segment elevations and elevated troponin I to 38 ng/mL with a normal B Type Natriuretic Peptide (BNP). On arrival to our ED, his repeat troponin was found to be 40 ng/mL. He had normal biventricular systolic function with normal coronary origins and size on transthoracic echocardiogram. He was admitted to the pediatric intensive care unit (PICU) for close monitoring due to severely elevated troponin and ECG changes. He was monitored with serial troponins every six hours and symptomatic management with nonsteroidal anti-inflammatory agents. His respiratory viral panel was negative for all the viruses. Due to the possibility of MIS-C, labs such as ferritin 128 ng/mL (normal), CRP 86.5 mg/L (elevated), d-dimers 0.75 mg/L (slightly elevated), and ESR 4 mm/hr (normal) were performed. His chest pain completely resolved, ST segment elevations improved, and troponins trended down within 24 hours to 11 ng/mL. Cardiac magnetic resonance imaging was planned to confirm the diagnosis of myocarditis but deferred due to the presence of vagal nerve stimulator in proximity to the heart.

Although his chest pain improved, the troponins plateaued between 11 and 13 ng/mL for another 24 hours with some diffuse T wave inversions on EKG. Computerized Tomographic Angiography (CTA) of the coronary arteries demonstrated a normal appearance of the coronary arteries without anomaly, stenosis, or occlusion. On PICU day #3, his troponins trended down to 4 ng/mL and he remained without chest pain. He was discharged home on ibuprofen with close follow-up with cardiology. He was evaluated in the outpatient cardiology office a week after discharge from PICU, where his repeat echocardiogram was normal and troponin I level of 0.17 ng/mL. He has planned follow-up in 6 weeks.

## 3. Case 2

A 17-year-old male with history of attention deficit hyperactivity disorder and mild intermittent asthma presented to a local urgent care center with chest pain. He received his second dose of Pfizer-BioNTech COVID-19 vaccine 3 days prior to presentation. Starting the day, he received his vaccine, he experienced fatigue, generalized body aches, headache, and fever of 101 F. He denied any cough, congestion, nausea, emesis, abdominal pain, or diarrhea. There was no known exposure or history of COVID-19 in the past. He described his chest pain as sharp, nonradiating, and constant pain localized to the sternum. It was exacerbated by deep inspiration but no change with movement or palpation. Additionally, he complained of shortness of breath with the chest pain. His labs showed an elevated troponin I of 5 ng/mL and ST segment elevations on the ECG concerning for myocarditis which prompted the transfer to our ED for cardiology evaluation. Echocardiogram showed mildly decreased left ventricular systolic function. He was admitted to the PICU for close monitoring due to his EKG changes and elevated troponin I. He also had negative viral respiratory panel, and his MIS-C labs included a normal ferritin and d-dimer with BNP of 121 pg/mL (mildly elevated) and CRP 95 mg/L (elevated), ESR 43 mm/hr (elevated), and negative SARS-CoV-2 IgG antibody. He was managed with serial troponins and supportive management with ibuprofen and narcotics for chest pain. He was transferred out of the PICU to the floor at hospital day 2 and then discharged home on day 3. His troponin level at the time of discharge is 0.55 ng/mL, and EKG showed normal ST segments. Follow-up is planned in 2-3 weeks.

## 4. Discussion

As per a report from Centers for Disease Control and Prevention, a total of 789 cases of myocarditis or pericarditis across all ages have been reported since April 2021 after the Pfizer/BioNTech and Moderna vaccines, most commonly after the second dose. After the FDA authorized the use of Pfizer-BioNTech COVID-19 vaccine in young people between ages 12 and 15 years of age in May 2021, federal agencies began receiving more reports about cases of chest pain or palpitations or other signs consistent with myocarditis or pericarditis in young adolescents. There were nearly 226 reports of myocarditis or pericarditis under age 30 years among which had been confirmed, and other 250 reports are still being investigated [3]. In those ages 16 to 17 years, there were 79 cases of myocarditis/pericarditis following 2.3 million administered second dose mRNA vaccinations [3]. The annual incidence of myocarditis from other causes in children and adolescents in United States is estimated to be 1-2 per 100,000 [7]. The number of these cases exceeds the cases expected in general unvaccinated children.

The cases that were reported to the vaccine adverse event reporting system (VAERS) have been predominantly males with a median age of 24 years following the second dose of the vaccine. The most common symptoms reported included chest pain, elevated cardiac enzymes, ST or T wave changes on ECG, dyspnea, and abnormal echocardiography/imaging. Both our patients presented with chest pain with elevated cardiac enzymes and ST segment changes on ECG. The echocardiograms in our patients appeared grossly normal with good biventricular systolic function. We ruled out acute COVID-19 infection as part of workup for the etiology of myocarditis in both our patients as there are many reports of acute myocarditis or pericarditis from COVID-19 infection both in the acute phase and delayed complication [7, 8]. Though children and young adolescents had been relatively spared from COVID-19 pulmonary manifestations, some of them succumbed to inflammatory syndrome called MIS-C proposed to be mediated by cytokine activation after several weeks of COVID-19 infection. Both our patients had reported no history of COVID-19 infection or known exposure to someone with COVID-19. The role of SARS-CoV-2 IgG antibodies in ruling out prior exposure in these cases is unknown due to vaccine-induced immunogenicity. Vaccines trigger antibodies to specific viral protein targets and currently authorized COVID-19 mRNA vaccines induce antibodies to the spike protein. If the patient had natural infection from COVID-19, the antibodies are produced to the nucleocapsid and spike proteins of the virus and at this time our SARS-CoV-2 antibody testing cannot differentiate the antibodies from either one making it difficult to differentiate the immunogenicity from vaccine or prior infection in our patients. Both our patients were ruled out for MIS-C due to low ferritin, d-dimer, fibrinogen, and no other clinical signs of multisystem involvement.

All patients presenting with chest pain, palpitations, shortness of breath, or syncope within 7 days from COVID-19 vaccination should get an ECG and troponin level to rule out postvaccine myocarditis. Patients with elevated troponins or ECG changes consistent with pericarditis or myocarditis should be admitted to the hospital with telemetry. These patients should be followed with serial troponin levels and ECGs. Echocardiogram should be obtained on admission to evaluate ventricular function, pericardial effusion, chamber enlargement, or valvular abnormalities. It should be repeated if there are any new arrhythmias or clinical deterioration. Elevated levels of cardiac enzymes such as cardiac troponin I can be seen in most patients with myocarditis, but the degree of elevation does not consistently correlate with disease severity as some patients with only mild ventricular dysfunction can have higher levels of troponin I than patients with moderate to severe ventricular dysfunction [9]. The highest level of troponin I that was reported by Marshall et al. [4] recently in their case series of seven adolescents with myocarditis after COVID-19 vaccine was 22 ng/mL. Our patient (case 1) had a level of 40 ng/mL which rapidly decreased to 11 ng/mL within 24 hours. Although troponin I is very specific marker for myocardial injury, there have been reports of troponin I elevation after a seizure [10, 11]. Since our patient has history of Lennox-Gastaut syndrome, he might have had some seizures contributing to such an elevation. It remains to be determined if the presence of copathologies in both our patients favored the increase in the troponin.

Extensive diagnostic evaluation for other infectious myocarditis etiologies should be performed in these patients. Infectious, particularly viral, etiologies are most common in children. The most common causes of viral myocarditis are enterovirus (coxsackie group B), adenovirus, parvovirus B19, Epstein-Barr virus, cytomegalovirus, and human herpes 6 [12]. These patients should be tested for SARS-CoV-2 PCR to rule out active COVID-19 infection. If the antibody testing can differentiate the vaccine-induced antibodies and infection-induced antibodies, then SARS-CoV-2 antibody testing is helpful to rule out prior infection as there are several reports of myocarditis or pericarditis as a part of MIS-C in children and young adolescents. Labs such as CRP, d-dimer, ferritin, and ESR should be sent in these patients to rule out MIS-C [13].

Endomyocardial biopsy (EMB) is considered the gold standard to confirm a clinical diagnosis of myocarditis but it has a fairly low sensitivity and is associated with considerable risk as it requires cardiac catheterization [14]. For these reasons, cardiac magnetic resonance imaging (CMRI) is increasingly used to diagnose myocarditis in children. CMRI can show myocardial edema, late gadolinium enhancement consistent with necrosis, or scarring and regional wall motion abnormalities [15]. Recent publication by Rosner et al. reported multifocal subepicardial late gadolinium enhancement in all their patients with myocarditis after COVID-19 vaccine and myocardial edema in 3 out of 7 patients [16]. CMRI was not performed in either of our patients as one of our patients (case 1) had a vagal nerve stimulator situated close to the heart and other patient (case 2) had resolving troponin leak within 24 hours. CMRI may be considered in patients with myocarditis from COVID-19 vaccine if the troponins are increasing and there is ventricular dysfunction on the echocardiogram to confirm the diagnosis of myocarditis.

The symptoms of vaccine-induced myocarditis mimic acute coronary syndromes. The incidence of acute coronary syndromes in children and young adolescents is very rare compared to middle-aged and elderly patients [17]. There have been multiple case reports of acute coronary syndrome associated with COVID-19 in adults who required invasive coronary angiography but no reports of coronary involvement in any patients who received COVID-19 vaccine so far [18]. Rosner et al. [16] performed coronary angiography in 3 out of 7 middle-aged patients with COVID-19 vaccine-associated myocarditis, and none of them revealed any obstructive coronary disease, and all of them were early or middle-aged patients. Marshall et al. [4] reported none of their adolescents required any coronary angiography in their case series. The role of cardiac catheterization in these adolescent patients with postvaccine myocarditis to rule out acute coronary syndrome is unclear at this time but considering the prevalence of acute coronary syndromes in this age group makes it of little or no benefit considering the risks associated.

In most cases that were reported, the clinical course of vaccine-associated myocarditis appears favorable as most of the patients have responded well to medications and rest with prompt improvement in symptoms with full recovery. Though the risk of myocarditis from vaccine seems higher than the general population, the benefit of COVID-19 vaccines clearly outweighs the risks given the potential morbidity of COVID-19 or any type of viral myocarditis. This problem is relatively new, and the CDC and its partners are actively monitoring reports and reviewing data and medical records to see if there is any association between the COVID-19 vaccine and myocarditis. The experience remains limited at this time regarding the investigations, management, and follow-up of this novel clinical entity. It is critical to report all these events to VAERS, and further studies are needed to explain the pathophysiological mechanism of this phenomenon so that these patients can be identified and treated appropriately. It is vital for all the health care providers taking care of adolescents to have knowledge about this phenomenon and make correct diagnosis in those presenting with chest pain after COVID-19 vaccine and in preventing unnecessary invasive procedures such as coronary angiogram to rule out acute coronary syndromes.

## 5. Patient Perspective

One of the patients shared that he was glad that the damage to the heart is temporary, and he was happy that he was able to go home in few days. He said that he did not regret taking the vaccine.

## Data Availability

The data used to support the findings of this study have been made available through the references and can be accessed by PubMed and other web-based resources.

## Supplementary Materials

Supplementary Materials CARE Case Report guideline checklist.
