# Supplementary material for: Acute Myopericarditis after COVID-19 Vaccine in Teenagers
Source: Case Rep Cardiol. 2021 Sep 20;2021:8268755. doi: 10.1155/2021/8268755 (PMC8476255; doi:10.1155/2021/8268755)
Supplement: Supplementary Materials — CARE Case Report guideline checklist. [file 8268755.f1.pdf]

CARE
case report guidelines

CARE Checklist of information to include when writing a case report

(cc) BY-NC-ND

✓

Topic	Item	Checklist item description	Reported on Line
**Title**	1	The diagnosis or intervention of primary focus followed by the words "case report"	Page 1 - Line 1
**Key Words**	2	2 to 5 key words that identify diagnoses or interventions in this case report, including "case report"	Page 2 - Line 10
**Abstract (no references)**	3a	Introduction: What is unique about this case and what does it add to the scientific literature?	Page 2 - Line 15-20
	3b	Main symptoms and/or important clinical findings	Page 3 - Lines 3-10
	3c	The main diagnoses, therapeutic interventions, and outcomes	Page 3 - Lines 3-10
	3d	Conclusion—What is the main "take-away" lesson(s) from this case?	Page 3 - Lines 14-20
**Introduction**	4	One or two paragraphs summarizing why this case is unique (**may include references**)	Page 4 - Lines 1-25
**Patient Information**	5a	De-identified patient specific information.	Page 5 Line 6, Page 6 line 10
	5b	Primary concerns and symptoms of the patient.	Page 5 - Line 9-12
	5c	Medical, family, and psycho-social history including relevant genetic information	N/A
	5d	Relevant past interventions with outcomes	None
**Clinical Findings**	6	Describe significant physical examination (PE) and important clinical findings.	Page 5 Line 13-17
**Timeline**	7	Historical and current information from this episode of care organized as a timeline	Page 5 & 6
**Diagnostic Assessment**	8a	Diagnostic testing (such as PE, laboratory testing, imaging, surveys).	Page 5 Line 15-25
	8b	Diagnostic challenges (such as access to testing, financial, or cultural)	Page 5 Line 23-25
	8c	Diagnosis (including other diagnoses considered)	Page 6 Line 1-3
	8d	Prognosis (such as staging in oncology) where applicable	N/A
**Therapeutic Intervention**	9a	Types of therapeutic intervention (such as pharmacologic, surgical, preventive, self-care)	N/A
	9b	Administration of therapeutic intervention (such as dosage, strength, duration)	N/A
	9c	Changes in therapeutic intervention (with rationale)	N/A
**Follow-up and Outcomes**	10a	Clinician and patient-assessed outcomes (if available)	Page 6 Line 5-7
	10b	Important follow-up diagnostic and other test results	Page 7 Line 2
	10c	Intervention adherence and tolerability (How was this assessed?)	N/A
	10d	Adverse and unanticipated events	N/A
**Discussion**	11a	A scientific discussion of the strengths AND limitations associated with this case report	Page 7 Line 17-24
	11b	Discussion of the relevant medical literature **with references**.	Page 7 Line 6-16
	11c	The scientific rationale for any conclusions (including assessment of possible causes)	Page 9 Line 23-25
	11d	The primary "take-away" lessons of this case report (without references) in a one paragraph conclusion	Page 10 Line 9-21
**Patient Perspective**	12	The patient should share their perspective in one to two paragraphs on the treatment(s) they received	Page 11 Line 1-2
**Informed Consent**	13	Did the patient give informed consent? Please provide if requested	Yes ☑ No ☐
